# Supplementary material for: Comparing Reproductive Health Awareness, Nutrition, and Hygiene among Early and Late Adolescents from Marginalized Populations of India: A Community-Based Cross-Sectional Survey
Source: Healthcare (Basel). 2021 Aug 2;9(8):980. doi: 10.3390/healthcare9080980 (PMC8394421; doi:10.3390/healthcare9080980)
Supplement: Supplementary file 1 [file healthcare-09-00980-s001.zip › healthcare-1272086-supplementary.pdf]

Table S1: Unadjusted logistic regression between eight outcomes and socio-demographic factors among adolescent girls (n=19164)

Variables	**Had anemia** (yes)[1] OR (95%CI)	**Consumed IFA tablets** (yes)[1] OR (95%CI)	**Heard about HIV/AIDS** (yes)[1] OR (95%CI)	**Heard about STI/RTI** (yes)[1] OR (95%CI)	**Open defecation** (yes)[1] OR (95%CI)	**Washed hands after defecation with soaps/ashes** (yes)[1] OR (95%CI)	**Had 3 or more meals in a day** (yes)[1] OR (95%CI)	**Safe menstrual hygiene practices** (yes)[1] OR (95%CI)
**Religion**								
Hindu	1.1(1.0,1.3)¶	1.2(1.1,1.3)*	1.1(1.0,1.2)§	1.2(1.1,1.3)*	2.5(2.2,2.8)*	0.5(0.4,0.6)*	1.3(1.2,1.4)*	0.4(0.4,0.5)*
Non-Hinduβ	*Reference*	*Reference*	*Reference*	*Reference*	*Reference*	*Reference*	*Reference*	*Reference*
**Caste**								
Non-marginalized	0.8(0.7,0.9)¶	2.0(1.8,2.2)*	1.4(1.3,1.6)*	0.6(0.5,0.7)*	0.07(0.06,0.09)*	3.8(3.1,4.5)*	1.1(1.0,1.3)¶	10.4(7.6,14.2)*
Other backward classes	0.6(0.6,0.7)*	0.9(0.9,1.0)	1.2(1.1,1.2)*	1.1(1.1,1.2)*	0.8(0.7,0.8)*	1.7(1.6,1.9)*	1.0(0.9,1.0)	1.4(1.2,1.6)*
Scheduled caste/tribes	*Reference*	*Reference*	*Reference*	*Reference*	*Reference*	*Reference*	*Reference*	*Reference*
**Socio-economic status**								
No cards	0.9(0.8,1.1)	1.0(0.9,1.1)	0.6(0.6,0.7)*	0.9(0.8,1.1)	0.6(0.6,0.7)*	0.6(0.5,0.7)*	1.0(0.9,1.2)	1.6(1.2,2.0)*
Above Poverty Line	0.7(0.6,0.7)*	1.2(1.1,1.3)*	0.8(0.7,0.8)*	0.8(0.8,0.9)§	0.5(0.4,0.5)*	1.1(1.0,1.2)§	0.5(0.5,0.6)*	0.8(0.7,0.9)§
Antayodya Ann Yojna† and Below Poverty Line	*Reference*	*Reference*	*Reference*	*Reference*	*Reference*	*Reference*	*Reference*	*Reference*
**Education status**								
Illiterate	1.4(1.1,1.7) §	0.4(0.3,0.5)*	0.2(0.1,0.2)*	0.4(0.3,0.5)*	2.5(2.1,3.0)*	0.1(0.1,0.2)*	1.5(1.2,2.0)§	0.2(0.1,0.3)*
Primary	0.6(0.6,0.8)*	0.5(0.5,0.6)*	0.1(0.1,0.2)*	0.3(0.3,0.4)*	1.5(1.3,1.7)*	0.4(0.4,0.5)*	0.9(0.8,1.1)	0.4(0.3,0.5)*
Upper primary	0.8(0.7,0.9)¶	0.8(0.7,0.8)*	0.3(0.2,0.3)*	0.4(0.3,0.5)*	1.2(1.1,1.3)*	0.7(0.6,0.8)*	1.0(0.9,1.1)	0.5(0.4,0.6)*
Secondary	0.9(0.8,1.0)	1.0(0.9,1.1)	0.7(0.6,0.7)*	0.7(0.6,0.8)*	0.9(0.8,1.0)	0.8(0.7,0.9)§	1.2(1.0,1.3)§	0.7(0.6,0.9)§
Senior secondary & above	*Reference*	*Reference*	*Reference*	*Reference*	*Reference*	*Reference*	*Reference*	*Reference*
**Areas**								
Rural	0.9(0.8,1.0)	0.6(0.5,0.6)*	0.9(0.8,1.0)	1.7(1.6,1.9)*	2986.3(420.4,21210.7)*	0.2(0.2,0.2)*	0.8(0.7,0.9)*	0.05(0.04,0.06)*
Urban	*Reference*	*Reference*	*Reference*	*Reference*	*Reference*	*Reference*	*Reference*	*Reference*

*p-value<0.001; §p-value<0.01; ¶p-value<0.05

βNon-Hindu includes Muslim, Buddhist, Jain, Sikhs, Christians, and Parsis; †*Antayodya Ann Yojna* includes extremely poor people.

[1]No was the reference category

Abbreviations: OR: Unadjusted Odds Ratio; CI: Confidence Interval; IFA: Iron Folic Acid tablets; RTI/STI: Reproductive Tract Infections/Sexually Transmitted Infections

Table S2: Adjusted logistic regression between eight outcomes and socio-demographic factors among adolescent girls (n=19162)

Variables	**Had anemia** (yes)[1] aOR (95%CI)	**Consumed IFA tablets** (yes)[1] aOR (95%CI)	**Heard about HIV/AIDS** (yes)[1] aOR (95%CI)	**Heard about STI/RTI** (yes)[1] aOR (95%CI)	**Open defecation** (yes)[1] aOR (95%CI)	**Washed hands after defecation with soaps/ashes** (yes)[1] aOR (95%CI)	**Had 3 or more meals in a day** (yes)[1] aOR (95%CI)	**Safe menstrual hygiene practices** (yes)[1] aOR (95%CI)
**Religion**								
Hindu	1.1(0.9,1.2)	1.4(1.3,1.6)*	1.1(1.0,1.2)¶	1.0(0.9,1.1)	1.5(1.3,1.7)*	0.8(0.7,0.9)§	1.4(1.3,1.6)*	0.9(0.7,1.1)
Non-Hindu[β]	*Reference*	*Reference*	*Reference*	*Reference*	*Reference*	*Reference*	*Reference*	*Reference*
**Caste**								
Non-marginalized	0.8(0.7,1.0)	1.6(1.5,1.8)*	1.5(1.4,1.7)*	0.8(0.7,0.9)§	0.3(0.2,0.3)*	1.9(1.6,2.3)*	1.1(1.0,1.3)¶	2.9(2.1,4.1)*
Other backward classes	0.7(0.6,0.8)*	0.9(0.8,1.0)	1.2(1.1,1.3)*	1.1(1.0,1.2)*	0.9(0.9,1.0)	1.6(1.4,1.7)*	1.1(1.0,1.2)§	1.3(1.2,1.5)*
Scheduled caste/tribes	*Reference*	*Reference*	*Reference*	*Reference*	*Reference*	*Reference*	*Reference*	*Reference*
**Socio-economic status**								
No cards	1.0(0.8,1.1)	0.8(0.7,0.9)¶	0.6(0.5,0.7)*	1.1(1.0,1.3)¶	1.2(1.0,1.4)§	0.4(0.3,0.4)*	1.0(0.8,1.2)	1.0(0.8,1.3)
Above Poverty Line	0.7(0.6,0.8)*	1.2(1.1,1.3)*	0.7(0.6,0.7)*	0.8(0.7,0.9)*	0.4(0.4,0.4)*	1.0(0.9,1.1)	0.5(0.5,0.6)*	0.7(0.6,0.8)*
Antayodya Ann Yojna[†] and Below Poverty Line	*Reference*	*Reference*	*Reference*	*Reference*	*Reference*	*Reference*	*Reference*	*Reference*
**Education status**								
Illiterate	1.5(1.2,1.8) §	0.5(0.4,0.6)*	0.2(0.2,0.3)*	0.4(0.3,0.6)*	2.0(1.6,2.4)*	0.2(0.1,0.2)*	1.6(1.2,2.0)*	0.3(0.2,0.4)*
Primary	1.0(0.9,1.2)	0.6(0.5,0.7)*	0.2(0.2,0.3)*	0.5(0.4,0.6)*	1.8(1.6,2.1)*	0.3(0.3,0.4)*	1.1(0.9,1.2)	0.4(0.3,0.5)*
Upper primary	1.2(1.1,1.4)§	0.8(0.8,0.9)¶	0.4(0.4,0.5)*	0.6(0.5,0.7)*	1.5(1.4,1.7)*	0.5(0.4,0.6)*	1.1(0.9,1.1)	0.4(0.3,0.5)*
Secondary	1.0(0.9,1.2)	1.0(0.9,1.1)	0.8(0.7,0.9)*	0.8(0.7,0.9)§	1.2(1.1,1.3)*	0.7(0.6,0.8)*	1.2(1.0,1.3)§	0.5(0.4,0.6)*
Senior secondary & above	*Reference*	*Reference*	*Reference*	*Reference*	*Reference*	*Reference*	*Reference*	*Reference*
**Age**								
Early adolescence (10-14)	0.4(0.4,0.5)*	0.8(0.7,0.9)*	0.5(0.4,0.5)*	0.5(0.4,0.5)*	0.9(0.8-0.9)¶	1.5(1.3,1.6)*	0.8(0.7-0.9)§	1.4(1.2,1.6)*
Late adolescence (15-19)	*Reference*	*Reference*	*Reference*	*Reference*	*Reference*	*Reference*	*Reference*	*Reference*
**Areas**								
Rural	0.8(0.7,0.9)§	0.6(0.5,0.6)*	0.9(0.8,1.0)	1.5(1.4,1.7)*	2437.3(342.9,17322.5)*	0.2(0.2,0.3)*	0.8(0.7,0.9)*	0.07(0.05,0.09)*
Urban	*Reference*	*Reference*	*Reference*	*Reference*	*Reference*	*Reference*	*Reference*	*Reference*

*p-value<0.001; §p-value<0.01; ¶p-value<0.05

[β]Non-Hindu includes Muslim, Buddhist, Jain, Sikhs, Christians, and Parsis; [†]*Antayodya Ann Yojna* includes extremely poor people.

[1]No was the reference category

Abbreviations: aOR: Adjusted Odds Ratio; CI: Confidence Interval; IFA: Iron Folic Acid tablets; RTI/STI: Reproductive Tract Infections/Sexually Transmitted Infections

Table S3: Unadjusted logistic regression between seven outcomes and socio-demographic factors among adolescent boys (n=19009)

Variables	**Had anemia** (yes)[1] OR (95%CI)	**Consumed IFA tablets** (yes)[1] OR (95%CI)	**Heard about HIV/AIDS** (yes)[1] OR (95%CI)	**Heard about STI/RTI** (yes)[1] OR (95%CI)	**Open defecation** (yes)[1] OR (95%CI)	**Washed hands after defecation with soaps/ashes** (yes)[1] OR (95%CI)	**Had 3 or more meals in a day** (yes)[1] OR (95%CI)
**Religion**							
Hindu	0.9(0.8,1.0)	0.4(0.4,0.5)*	1.2(1.1,1.3)*	1.8(1.6,2.0)*	4.8(4.1,5.5)*	0.5(0.4,0.5)*	1.3(1.2,1.4)*
Non-Hindu[β]	*Reference*	*Reference*	*Reference*	*Reference*	*Reference*	*Reference*	*Reference*
**Caste**							
Non-marginalized	1.6(1.4,1.9)*	2.6(2.4,2.9)*	1.4(1.2,1.5)*	0.5(0.5,0.6)*	0.1(0.1,0.1)*	2.2(1.9,2.6)*	1.2(1.0,1.3)[§]
Other backward classes	0.6(0.5,0.7)*	0.5(0.5,0.6)*	1.1(1.0,1.1)[§]	1.0(0.9,1.1)	1.0(0.9,1.1)	1.3(1.2,1.5)*	1.1(1.0,1.2)*
Scheduled caste/tribes	*Reference*	*Reference*	*Reference*	*Reference*	*Reference*	*Reference*	*Reference*
**Socio-economic status**							
No cards	1.1(0.9,1.4)	1.3(1.2,1.5)*	0.6(0.5,0.6)*	0.8(0.7,1.0)	0.8(0.7,0.9)*	0.6(0.5,0.7)*	1.0(0.8,1.1)
Above Poverty Line	0.7(0.6,0.8)*	1.3(1.2,1.4)*	0.7(0.7,0.8)*	0.6(0.6,0.7)*	0.4(0.3,0.4)*	1.2(1.1,1.4)*	0.5(0.5,0.6)*
Antayodya Ann Yojna[†] and Below Poverty Line	*Reference*	*Reference*	*Reference*	*Reference*	*Reference*	*Reference*	*Reference*
**Education status**							
Illiterate	2.0(1.2,3.3)[§]	0.9(0.6,1.3)	0.2(0.2,0.3)*	0.3(0.2,0.4)*	2.0(1.5,2.8)*	0.3(0.2,0.5)*	0.8(0.6,1.1)
Primary	1.5(1.1,2.1)[§]	2.4(2.0,2.8)*	0.2(0.1,0.2)*	0.3(0.2,0.3)*	1.8(1.5,2.2)*	0.4(0.3,0.5)*	1.1(1.0,1.3)[¶]
Upper primary	1.8(1.3,2.4)*	2.7(2.3,3.3)*	0.5(0.4,0.6)*	0.4(0.3,0.5)*	1.4(1.2,1.7)*	0.6(0.4,0.7)*	1.3(1.1,1.6)*
Secondary	1.5(1.1,2.2)[§]	2.1(1.8,2.5)*	0.8(0.7,1.0)	0.6(0.5,0.7)*	1.1(0.9,1.4)	0.7(0.5,1.0)	1.0(0.8,1.2)
Senior secondary & above	*Reference*	*Reference*	*Reference*	*Reference*	*Reference*	*Reference*	*Reference*
**Areas**							
Rural	0.3(0.3,0.4)*	0.07(0.06,0.08)*	0.8(0.7,0.8)*	1.7(1.6,1.9)*	853.2(213.2,3414.4)*	0.4(0.3,0.4)*	0.6(0.5,0.6)*
Urban	*Reference*	*Reference*	*Reference*	*Reference*	*Reference*	*Reference*	*Reference*

*p-value<0.001; [§]p-value<0.01; [¶]p-value<0.05

[β]Non-Hindu includes Muslim, Buddhist, Jain, Sikhs, Christians, and Parsis; [†]*Antayodya Ann Yojna* includes extremely poor people.

[1]No was the reference category

Abbreviations: OR: Unadjusted Odds Ratio; CI: Confidence Interval; IFA: Iron Folic Acid tablets; RTI/STI: Reproductive Tract Infections/Sexually Transmitted Infections

Table S4: Adjusted logistic regression between seven outcomes and socio-demographic factors among adolescent boys (n=19008)

Variables	**Had anemia** (yes)[1] aOR (95%CI)	**Consumed IFA tablets** (yes)[1] aOR (95%CI)	**Heard about HIV/AIDS** (yes)[1] aOR (95%CI)	**Heard about STI/RTI** (yes)[1] aOR (95%CI)	**Open defecation** (yes)[1] aOR (95%CI)	**Washed hands after defecation with soaps/ashes** (yes)[1] aOR (95%CI)	**Had 3 or more meals in a day** (yes)[1] aOR (95%CI)
**Religion**							
Hindu	1.1(0.9,1.3)	0.7(0.6,0.8)*	1.2(1.1,1.3)*	1.5(1.3,1.7)*	3.5(3.0,4.1)*	0.6(0.5,0.7)*	1.5(1.3,1.6)*
Non-Hindu[β]	*Reference*	*Reference*	*Reference*	*Reference*	*Reference*	*Reference*	*Reference*
**Caste**							
Non-marginalized	1.2(1.0,1.4)¶	0.9(0.8,1.0)	1.2(1.1,1.3)*	0.6(0.5,0.8)*	0.3(0.3,0.4)*	1.4(1.2,1.7)*	1.0(0.9,1.2)
Other backward classes	0.7(0.6,0.8)*	0.5(0.4,0.5)*	1.0(1.0,1.1)¶	1.0(0.9,1.1)	1.3(1.2,1.4)*	1.2(1.1,1.3)*	1.3(1.2,1.4)*
Scheduled caste/tribes	*Reference*	*Reference*	*Reference*	*Reference*	*Reference*	*Reference*	*Reference*
**Socio-economic status**							
No cards	0.9(0.7,1.1)	0.6(0.5,0.7)*	0.6(0.5,0.7)*	1.1(0.9,1.3)	1.3(1.1,1.5)§	0.5(0.4,0.6)*	0.9(0.7,1.0)
Above Poverty Line	0.8(0.7,0.9)§	2.0(1.9,2.2)*	0.7(0.6,0.7)*	0.6(0.5,0.6)*	0.3(0.3,0.3)*	1.2(1.1,1.3)*	0.5(0.5,0.6)*
Antayodya Ann Yojna[†] and Below Poverty Line	*Reference*	*Reference*	*Reference*	*Reference*	*Reference*	*Reference*	*Reference*
**Education status**							
Illiterate	1.6(1.0,2.7)¶	0.4(0.3,0.6)*	0.3(0.2,0.4)*	0.3(0.2,0.5)*	2.5(1.7,3.5)*	0.3(0.2,0.4)*	0.7(0.5,1.0)
Primary	1.3(0.9,1.8)	1.0(0.8,1.2)	0.3(0.2,0.3)*	0.4(0.3,0.5)*	2.7(2.2,3.2)*	0.3(0.2,0.4)*	1.1(0.9,1.3)
Upper primary	1.4(1.0,1.9)¶	1.5(1.2,1.8)*	0.5(0.5,0.6)*	0.5(0.4,0.6)*	1.9(1.5,2.3)*	0.5(0.4,0.6)*	1.3(1.0,1.5)§
Secondary	1.3(0.9,1.8)	1.6(1.3,1.9)*	0.8(0.7,1.0)	0.6(0.5,0.8)*	1.3(1.1,1.7)§	0.7(0.5,0.9)¶	1.0(0.8,1.2)
Senior secondary & above	*Reference*	*Reference*	*Reference*	*Reference*	*Reference*	*Reference*	*Reference*
**Age**							
Early adolescence (10-14)	0.8(0.7,0.9)¶	1.6(1.5,1.7)*	0.5(0.4,0.5)*	0.6(0.5,0.6)*	0.9(0.8,1.0)	1.3(1.2,1.5)*	1.0(0.9,1.1)
Late adolescence (15-19)	*Reference*	*Reference*	*Reference*	*Reference*	*Reference*	*Reference*	*Reference*
**Areas**							
Rural	0.4(0.3,0.5)*	0.06(0.06,0.07)*	0.6(0.6,0.7)*	1.4(1.2,1.6)*	761.6(190.1,3051.1)*	0.4(0.3,0.4)*	0.6(0.5,0.6)*
Urban	*Reference*	*Reference*	*Reference*	*Reference*	*Reference*	*Reference*	*Reference*

*p-value<0.001; §p-value<0.01; ¶p-value<0.05

[β]Non-Hindu includes Muslim, Buddhist, Jain, Sikhs, Christians, and Parsis; [†]*Antayodya Ann Yojna* includes extremely poor people.

[1]No was the reference category

Abbreviations: aOR: Adjusted Odds Ratio; CI: Confidence Interval; IFA: Iron Folic Acid tablets; RTI/STI: Reproductive Tract Infections/Sexually Transmitted Infections;
